# Relevance of Somatosensory Evoked Potential Amplitude After Cardiac Arrest

**DOI:** 10.3389/fneur.2020.00335

**Published:** 2020-04-28

**Authors:** Astrid B. Glimmerveen, Hanneke M. Keijzer, Barry J. Ruijter, Marleen C. Tjepkema-Cloostermans, Michel J. A. M. van Putten, Jeannette Hofmeijer

**Affiliations:** ^1^Department of Neurology, Rijnstate Hospital, Arnhem, Netherlands; ^2^Department of Intensive Care Medicine and Neurology, Donders Institute for Brain Cognition, and Behaviour, Radboud University Medical Center, Nijmegen, Netherlands; ^3^Clinical Neurophysiology, Technical Medical Centre, University of Twente, Enschede, Netherlands; ^4^Department of Neurology and Clinical Neurophysiology, Medisch Spectrum Twente, Enschede, Netherlands

**Keywords:** SSEP, EEG, prognosis, postanoxic coma, cardiac arrest

## Abstract

**Objective:** We present relations of SSEP amplitude with neurological outcome and of SSEP amplitude with EEG amplitude in comatose patients after cardiac arrest.

**Methods:** This is a *post hoc* analysis of a prospective cohort study in comatose patients after cardiac arrest. Amplitude of SSEP recordings obtained within 48–72 h, and EEG patterns obtained at 12 and 24h after cardiac arrest were related to good (CPC 1-2) or poor (CPC 3-5) outcome at 6 months. In 39% of the study population multiple SSEP measurements were performed. Additionally, SSEP amplitude was related to mean EEG amplitude.

**Results:** We included 138 patients (77% poor outcome). Absent SSEP responses, a N20 amplitude <0.4 μV within 48–72 h, and suppressed or synchronous EEG with suppressed background at 12 or 24 h after cardiac arrest were invariably associated with a poor outcome. Combined, these tests reached a sensitivity for prediction of poor outcome up to 58 at 100% specificity. N20 amplitude increased with a mean of 0.55 μV per day in patients with a poor outcome, and remained stable with a good outcome. There was no statistically significant correlation between SSEP and EEG amplitudes in 182 combined SSEP and EEG measurements (*R*^2^ < 0.01).

**Conclusions:** N20 amplitude <0.4 μV is invariably associated with poor outcome. There is no correlation between SSEP and EEG amplitude.

**Significance:** SSEP amplitude analysis may contribute to outcome prediction after cardiac arrest.

## Introduction

Approximately half of all comatose patients after cardiac arrest never regain consciousness as a result of severe post anoxic encephalopathy (1, 2). Early identification of patients without a chance of recovery of brain functioning may prevent continuation of futile treatment and save costs (3).

Bilaterally absent somatosensory evoked potentials (SSEPs) after electrical stimulation of the median nerve at the wrist is strongly associated with a poor outcome and has been included in prognostication guidelines since the late 1990s (4). If tested during normothermia, off sedation, and >24 h after cardiac arrest, the specificity of absent SSEP recordings for prediction of poor outcome was 98–100%. Possibly, SSEP recordings as early as 12 h after cardiac arrest can predict poor outcome with 100% specificity, though the effect of targeted temperature management (TTM) at this early timepoint has not been investigated (5). The sensitivity of SSEP for detection of patients with a poor outcome is low to moderate, varying from 24 to 46% (6–10).

Evolving electroencephalography (EEG) patterns have been related to outcome of comatose patients after cardiac arrest. Suppressed and synchronous patterns with suppressed background ≥12 h after cardiac arrest were invariably associated with a poor outcome (5, 8, 9, 11–13). Otherwise, EEG patterns with continuous background at ≥20 μV within 12 h after cardiac arrest were strongly related to good neurological recovery (8, 9, 11, 14, 15).

Low EEG amplitudes are consistently associated with poor outcome. However, only few studies addressed the role of SSEP amplitude. Two studies show that an early N20 amplitude <0.65 μV (16) or <0.62 μV (17) reliably predicts poor outcome, even when tested during treatment with sedative drugs or hypothermia. However, others stress that low or even absent SSEP responses can be found during treatment with hypothermia in patients with a good recovery (18). Next to low amplitudes, giant SSEP responses, defined as N20 amplitude from 15 up to 50 μV, have also been associated with poor outcome (19). In a small case study giant SSEPs were suggested to be associated with paroxysmal activity on EEG and associated with poor outcome in anoxic brain injury (20). However, more recently, giant SSEPs defined as N20-P24 peak-to-peak amplitudes >5.5 μV were considered uncommon in post anoxic encephalopathy with paroxysmal activity on EEG (21).

In the current study, we analyze the association between SSEP amplitude and outcome in comatose patients after cardiac arrest. Analogous to the EEG, we hypothesize that a SSEP amplitude below a certain value is invariably associated with poor outcome. In addition, we study the association between SSEP and EEG amplitudes.

## Materials and Methods

### Study Design and Patients

This is a *post hoc* analysis of data collected within a multicenter prospective cohort study on EEG based outcome prediction of comatose patients after cardiac arrest on intensive care units (ICUs) of two teaching hospitals. Patients were included between 2010 and 2011 in Medisch Spectrum Twente (MST) and between 2012 and 2018 in Rijnstate Hospital. Consecutive, adult, comatose patients after cardiac arrest (Glasgow Coma Scale score ≤ 8), admitted to the ICU, were included. Exclusion criteria were concomitant acute stroke, traumatic brain injury, preexisting dependency in daily life, or progressive neurodegenerative disease. EEG registration was performed in all patients.

### Treatment

“Patients were treated according to standard protocols for comatose patients after cardiac arrest. This included targeted temperature management at 33 or 36°C. In Rijnstate Hospital the target temperature was set from 33 to 36°C in February 2014. In Medisch Spectrum Twente, there was a gradual shift from 33 to 36°C in 2017. Target temperature was induced as soon as possible after arrival at the emergency room or ICU and maintained for 24 h. After 24 h, passive re-warming was controlled to a speed of 0.25 or 0.5°C per hour. In case of T>38°C in combination with a Glascow Coma Scale score ≤8, targeted temperature management was restarted at 36.5–37.5°C for another 48 h. In Medisch Spectrum Twente, propofol and fentanyl or remifentanil were used for sedation. In Rijnstate Hospital, patients received a combination of propofol, midazolam, and/or morphine. Mostly, analgo-sedation was discontinued at a body temperature of 36.5°C. In both hospitals, a non-depolarizing muscle relaxant (rocuronium or atracurium) was occasionally added in case of severe compensatory shivering.

Withdrawal of treatment was considered at >72 h, during normothermia, and off sedation. Withdrawal of treatment based on severity and prognosis of postanoxic encephalopathy was never before 72 h.

Decisions on treatment withdrawal were based on international guidelines including incomplete return of brainstem reflexes, treatment-resistant myoclonus, and bilateral absence of evoked SSEPs.” (9). The EEG within 72 h or low amplitude SSEP were not taken into account.

### Outcome

“The primary outcome measure was neurological outcome expressed as the score on the five-point Glasgow-Pittsburgh Cerebral Performance Category (CPC) (22). Outcome was dichotomized as “good” (CPC 1-2) or “poor” (CPC 3-5). CPC scores were obtained by telephone follow-up 6 months after cardiac arrest by one of the investigators (MTC, BR, or HK), blinded for EEG patterns and SSEP recordings. Scoring was based on a Dutch translation of the EuroQol-6D questionnaire.” (9).

### SSEP Recordings and Analyses

In the first 47 patients included at the MST, repeated SSEP recordings during the first 5 days of the ICU stay, or until discharge from the ICU, were intended. For patients in Rijnstate Hospital and later patients in MST, SSEP recordings were performed at the discretion of the treating physician. Generally, SSEPs were recorded between 48 and 72 h in patients that remained comatose after restoration of normothermia and off-sedation. SSEP recordings were made using a Nicolet Bravo system (Viasys, Houten, The Netherlands). “The SSEP was measured after stimulation of the right and left median nerve using a bipolar surface electrode at the wrist. Stimulus duration was set at 0.3 ms and stimulus amplitude was adjusted until a visible twitch of the thumb was produced. Two sets of >200–1000 responses were averaged, band pass filtered between 0.1 Hz and 2.5 kHz, and notch filtered around 50 Hz. Stimulus frequency was set at 1.7 or 2.3 Hz. Silver-silver chloride cup electrodes were placed at Erb's point, cervical spine (C5), and 2 cm posterior to C3 and C4 (C3' and C4'). Fz was used as a reference.” (8). “Skin-electrode impedance was kept below 5 kΩ. Peripheral N9 and N13 responses, and cortical N20 and P25 responses were recorded. SSEP results were categorized as present or absent based on the presence or absence of N20 responses.” (10). Unilateral or bilateral presence of the N20 response was considered as present SSEP. SSEP recordings were classified as inconclusive if noise level was higher than signal or in the absence of peripheral responses. The SSEP amplitude was defined as the N20/P25 peak-to-peak difference.

We evaluated the relation between SSEP amplitude and outcome and the relation between SSEP amplitude and EEG amplitude. If patients had more than one SSEP recording, we used the measurement closest to 48 h after cardiac arrest for this analysis. Patients with single SSEP measurements were typically measured between 48 and 72 h after cardiac arrest. SSEP measurements performed within 24 h after cardiac arrest were excluded for these analyses.

In addition, we studied the evolution of the N20 amplitude over time in patients with repeated SSEP measurements. SSEP measurements on day one were acquired during hypothermia.

### EEG Measurements and Analyses

Continuous EEG recordings were started as soon as possible after arrival at the ICU and continued for at least 3 days, or until discharge from the ICU. Twenty-one silver–silver chloride cup electrodes were placed on the scalp according to the international 10–20 system. A Neurocenter EEG recording system (Clinical Science Systems, the Netherlands) or a Nihon Kohden system (VCM Medical, the Netherlands) was used. All EEG analyses were prespecified and performed offline. EEG analyses were performed visually, at 12 and 24 h after cardiac arrest, and at the time of SSEP recordings ±1 h. EEG epochs of 5 min were automatically selected by a computer algorithm. These epochs were presented to a reviewer by the computer, in random order. Epochs were analyzed and classified by two reviewers, independently. Upon disagreement, consensus was determined, where a third reviewer was consulted, when necessary. The EEG was categorized in the following patterns: suppression (< 10 μV), low voltage (10–20 μV), synchronous patterns with suppressed background (defined as burst suppression with identical bursts, burst suppression with generalized, abrupt onset bursts with suppressed background, or GPDs with suppressed background), other burst suppression patterns, GPDs with other background, other epileptiform patterns and continuous patterns (9, 23, 24).

The mean amplitude of the 5 min EEG epochs at the time of SSEP recordings was estimated based on bipolar channels C3-Fz and C4-Fz, because these approach the SSEP montage C3'-Fz and C4'-Fz. The EEG amplitude was defined as the mean deviation from zero, after application of a 4th order band pass Butterworth filter between 0.5 and 30 Hz. A sliding window of 1 second with 50% overlap was applied. The maximum amplitude of C3-Fz and C4-Fz was defined for each window. These amplitudes were averaged to get a mean amplitude for both channels per 5 min epoch.

### Statistical Analyses

Demographic, baseline, EEG, and SSEP data are presented in a descriptive way. Differences between groups of patients were tested by a chi-square test or Fisher's exact test for categorical variables and Student's *t*-test for continuous variables, given a normal distribution, or Mann–Whitney *U*-test. Sensitivity, specificity, positive predictive value (PPV), and negative predictive value (NPV) were calculated for (groups of) predictors of poor or good outcome, including corresponding 95% confidence intervals. Evolution of N20 SSEP amplitudes over time was tested using regression analysis. Analyses were performed with SPSS-25 (IBM), Mathlab (version 2018,The MathWorks, Inc.), and GraphPad (version 5.03, Prism8).

### Data Availability Statement

Raw EEG and outcome data used for this article will be kept for at least 15 years and made available upon request, for verification of results or new relevant research questions, conditionally. Conditions include optimal data safety, adequate methodology, mutual appointments on collaboration, and approval of all collaborators.

## Results

**Figure 1:**
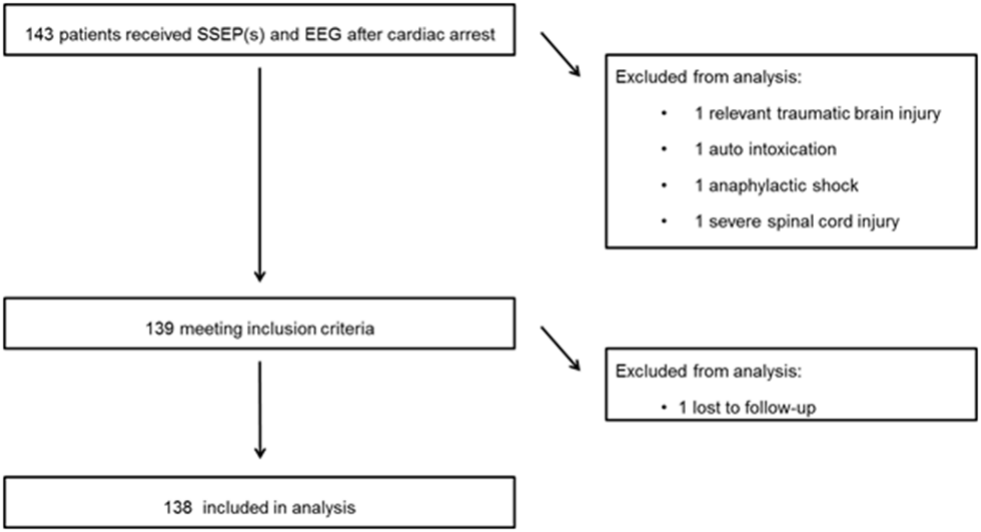
Flow of patients through this study. SSEP, somatosensory evoked potential; EEG, electroencephalogram.

Continuous EEG recordings were started in 395 comatose patients after cardiac arrest in the ICU. Of these, 143 patients underwent one or more SSEP recordings. Four patients were excluded from this analysis for the following reasons: relevant traumatic brain injury (1), auto intoxication (1), anaphylactic shock (1), severe spinal cord injury (1) (Figure 1). One subject was lost to follow up, leaving 138 patients for analyses (54 in MST and 85 in Rijnstate Hospital). Baseline characteristics are summarized in Table 1. For 2 patients, we used the CPC score at 3 months because they were lost to follow-up at 6 months. Poor neurological outcome occurred in 106 patients (77%), of whom 97 died.

**Table 1:** Patient characteristics and differences between patients with good and poor neurological outcome.

	**Good outcome** ***N* = 32**	**Poor outcome** ***N* = 106**	***p*-value**
Female sex	14/32 (44%)	20/106 (19%)	<0.01
Mean age (±SD)	65.9 ± 11	66.7 ± 11.2	0.9
OHCA	30/32 (94%)	92/106 (87%)	0.4
Cardiac etiology	28/29 (97%)	74/93 (80%)	0.2
Shockable rhythm (VF or VT)	31/32 (97%)	67/106 (63%)	<0.01
Mild therapeutic hypothermia (33°C)^*^	27/32 (84%)	54/105 (51%)	<0.01
Propofol in first 24 h	30/31 (97%)	84/102 (82%)	0.05
Max propofol dose in first 24 h (mg/kg/h)	2.62 ± 1.10	2.59 ± 1.06	0.9
Midazolam in first 24 h	8/31 (26%)	37/102 (36%)	0.4
Max midazolam dose in first 24 h (μg/kg/h)	166.9 ± 82.4	165 ± 90.2	0.7
Suppressed, or synchronous patterns on suppressed background at 12 h	0/25 (0%)	39/72 (54%)	<0.01
Suppressed, or synchronous patterns on suppressed background at 24 h	0/30 (0%)	32/93 (34%)	<0.01
Bilaterally absent SSEP^**^	0/31 (0%)	41/98 (42%)	<0.01
Continuous EEG at 12 h	6/25 (24%)	2/64 (3%)	<0.01

*SD, standard deviation; OHCA, out of hospital cardiac arrest; VF, ventricular fibrillation; unfavorable, suppressed, low-voltage, or synchronous patterns with suppressed background; SSEP, somatosensory evoked potential; unknown variables were treated as missing variables*.

* *The remainder of patients was treated at 36°C*.

** *Bilaterally absent SSEP generally between 48–72 h, never within 24 h after cardiac arrest and only 1 measurement per patient*.

### SSEP in Relation to Outcome

Bilateral absent SSEP responses were observed in 41 patients, who all had a poor outcome. Predictive values are summarized in Table 2.

**Table 2:** Predictive values of (combinations of) EEG and SSEP parameters.

**Predictor**	**Predicted outcome**	**Specificity** **(95% CI)**	**Sensitivity** **(95% CI)**	**PPV** **(95% CI)**	**NPV** **(95% CI)**
Continuous EEG pattern at 12 h	Good	98% (92–100)	19% (8–37)	75% (36–96)	80% (72–86)
N20 > 3.6 μV^*^	Good	96% (90–99)	31% (17–50)	71% (42–90)	82% (74–88)
Absent SSEP N20^*^	Poor	100% (87–100)	39% (30–49)	100% (89–100)	33% (24–44)
0<N20<0.4 μV ^*^	Poor	100% (87–100)	11% (6–20)	100% (70–100)	25% (18–34)
Suppressed or synchronous EEG on suppressed background at 12 h	Poor	100% (87–100)	37% (28–47)	100% (89–100)	32% (24–43)
Suppressed or synchronous EEG on suppressed background at 24 h	Poor	100% (87–100)	30% (22–40)	100% (87–100)	30% (22–40)
Suppressed or synchronous EEG on suppressed background at 12 h or N20 < 0.4 μV	Poor	100% (87–100)	58% (48–67)	100% (93–100)	42% (31–54)
Suppressed or synchronous EEG on suppressed background at 24 h or N20 < 0.4μV	Poor	100% (87–100)	53% (43–63)	100% (92–100)	39% (29–51)

*CI, confidence interval; SSEP, somatosensory evoked potential*.

* *Timing SSEP generally between 48 and 72 h, never within 24 h after cardiac arrest*.

In case of a preserved SSEP response, N20 amplitudes ranged between 0.03 and 17 μV at C4' and between 0.10 and 10 μV at C3'. In patients with a poor outcome, N20 amplitudes ranged between 0.03 and 17 μV, in patients with a good outcome between 0.4 and 11 μV. N20 < 0.4 μV was found in 12 patients with a poor outcome and 0 patients with a good outcome. Five patients with N20 < 0.4 μV had suppressed or synchronous patterns on suppressed background at 12 h after cardiac arrest. Seven had other EEG patterns. Four patients with N20 < 0.4 μV had suppressed or synchronous patterns on suppressed background EEG at 24 h after cardiac arrest. Eight had other EEG patterns. Bilaterally N20 amplitudes > 3.60 μV were found in 10 patients with good outcome. Two of these had N20 amplitudes > 5.5 μV. Four patients with poor outcome had bilaterally N20 amplitudes > 3.60 μV. Three of these had N20 amplitudes >5.5 μV.

### EEG in Relation to Outcome

Suppressed or synchronous patterns on a suppressed background EEG at 12 and 24h was observed in 39 and 32 patients, respectively, and were invariably associated with a poor outcome. Preserved N20 SSEP responses were found in 9 of these 39 patients at 12 h and in 7 of these 32 patients at 24 h.

### Repeated SSEP

In 47 patients, SSEP recordings were performed repeatedly during the first five days after cardiac arrest. All these patients were treated with mild therapeutic hypothermia. Eight patients had two daily SSEP recordings on day 1-2, 14 patients had three daily SSEP recordings on day 1-3, 10 patients had four daily SSEP recordings on day 1-4, and 15 patients had five daily SSEP recordings on day 1-5. One patient had preserved responses at the first four recordings and absent responses at the fifth SSEP recording. In all other patients with repeated SSEP recordings, N20 responses were either present (*n* = 41), or absent (*n* = 5).

**Figure 2:**
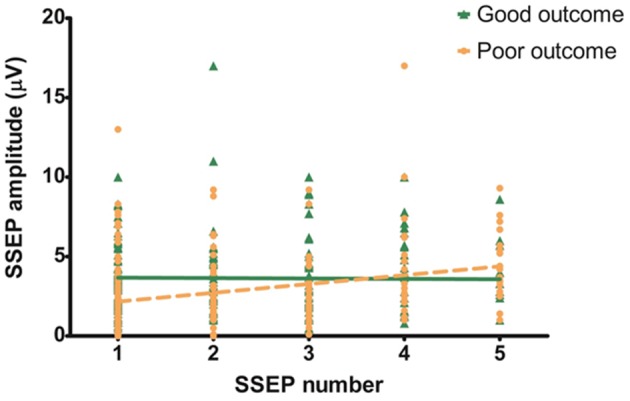
Evolution of N20 amplitude over time. Slope of the good outcome groups is not significantly different from zero (−0.03 μV; 95% CI: −0.30–0.25; *P* < 0.05; R2: 0.00). Slope of the poor outcome group shows a statistically significant increase (0.55 μV; 95% CI: 0.33–0.78; *P* = 0.86; R2: 0.09).

In the poor outcome group there was a mean increase in amplitude of 0.55 μV per SSEP measurement, in the good outcome group the amplitude was stable (−0.03 μV). The increase of amplitude in the poor outcome group was statistically significant (*P* < 0.05, Figure 2) with a R^2^ of 0.09. The change in amplitude in the good outcome group was not statistically significant (*P* = 0.86, Figure 2) with a R^2^ of 0.00.

### SSEP Amplitude in Relation to EEG Amplitude

**Figure 3:**
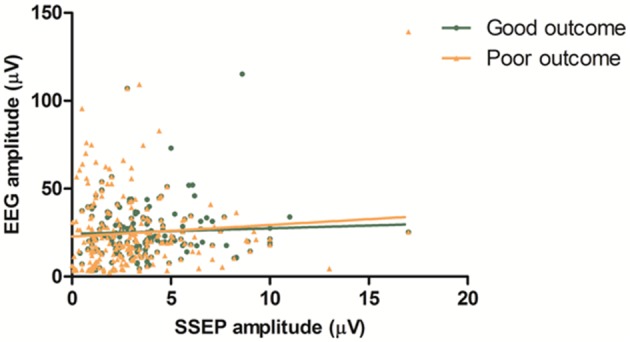
EEG vs. SSEP amplitude. Mean amplitudes of left and right hemisphere are combined in this figure. There is no statistically significant correlation between SSEP amplitude and EEG amplitude (*R*^2^ < 0.01). There is no statistically significant difference of SSEP/EEG amplitude ratio between patients with good and poor outcome (*P* = 0.64).

We related the mean amplitude of EEG to the amplitude of the SSEP. Based on visual analysis, seven EEG epochs (6 patients) were excluded from this analysis because of artifacts. Thus, 182 combined EEG and SSEP measurements were included. There was no statistically significant correlation between SSEP amplitude and EEG amplitude (*R*^2^ < 0.01). There was no statistically significant difference of SSEP/EEG amplitude ratio between patients with good and poor outcomes (*P* = 0.64, Figure 3).

## Discussion

We confirm that SSEP amplitude includes relevant information for outcome prediction of comatose patients after cardiac arrest. A N20 amplitude <0.4 μV was invariably associated with poor outcome, whereas bilaterally N20 >3.60 μV was associated with good outcome. Predictive values of SSEP may increase by taking the amplitude into account. We found no correlation between SSEP and EEG amplitude. We found no additional value of repeated SSEP measurements during the first five days after cardiac arrest.

Our results confirm earlier findings on the relevance of SSEP amplitude for outcome prediction of comatose patients after cardiac arrest. However, these studies on SSEP amplitude reported higher cut off values for poor outcome prediction of approximately 0.6 μV (16, 17). In these previous studies, SSEP was often recorded very early, during treatment with targeted temperature management and sedation. We used SSEP recordings at normothermia, off-sedation and >24 h after cardiac arrest for outcome prediction, and observed that N20 amplitudes tend to increase over time in patients with a poor outcome. However, despite this increase in N20 amplitude, once an amplitude <0.4 μV had been observed, this was invariably associated with poor outcome according to our data. Future research is needed to validate the reported threshold values.

The lack of correlation between SSEP amplitude and EEG amplitude probably reflects a difference in pathophysiology of disturbed SSEP and EEG patterns. SSEP depends on thalamocortical projections (25) whereas EEG rhythms primarily reflect pyramidal cell functioning, including their synchrony, for which a sufficient number of functioning pyramidal synapses is required (26).

Another possible explanation could be that high amplitude bursts on a suppressed background may have been averaged out due to the windowing approach. Results might be different when for example only the highest amplitude within a 5 min interval is used. However, this method would be more sensitive to high amplitude artifacts in the EEG signal.

Our study has certain limitations. SSEP measurements were mostly performed on request by the treating physician, when patients did not regain consciousness after withdrawal of sedation. This has likely introduced selection bias toward a subgroup of comatose patients after cardiac arrest with relatively severe encephalopathy. This is reflected by the high incidence of a poor outcome of 77% and has contributed to the high sensitivity of SSEP for poor outcome prediction. Of note, in the first 47 patients in the MST (39% of the complete data set) daily SSEP measurements were done until discharge from the ICU. Second, a potential problem in unblinded studies investigating diagnostic accuracy is the self-fulfilling prophecy. This characterizes almost all studies on this topic (27). Although EEG classifications were assigned offline, and treatment withdrawal was never based on the EEG or low SSEP amplitude, attending physicians were not blinded for the SSEP outcome. Absence of N20 responses during normothermia and in absence of sedative medication was generally followed by withdrawal of life sustaining treatment. In a recent study, life sustaining treatment was continued in case of absent SSEP responses, thereby eliminating the effect of self-fulfilling prophecy. They still found a 100% specificity in prediction of poor outcome for absent SSEP responses and poor EEG at 12 h, which indicates that the risk of misclassification based on SSEP recording is probably small (5).

## Conclusion

N20 amplitude <0.4 μV reliably predicts poor outcome of comatose patients after cardiac arrest. There is no correlation between SSEP and EEG amplitude.

## Data Availability Statement

The datasets generated for this study are available on request to the corresponding author.

## Ethics Statement

The studies involving human participants were reviewed and approved by Medical Ethics Committee Twente. Written informed consent for participation was not required for this study in accordance with the national legislation and the institutional requirements.

## Author Contributions

AG design and conceptualization of the study, data collection, data analysis and interpretation, statistical analyses, and drafted the manuscript. JH and MP design and conceptualization of the study, data collection, data analysis and interpretation, and revising the manuscript for intellectual content. HK design and conceptualization of the study, data collection, data interpretation, statistical analyses, and revising the manuscript for intellectual content. MT-C and BR data interpretation, data collection, and revising the manuscript for intellectual content.

## Conflict of Interest

MP is co-founder of Clinical Science Systems, which is a supplier of EEG systems for one of the participating sites (Medisch Spectrum Twente). Clinical Science Systems did not provide funding and was not involved in the design, execution, analysis, interpretation or publication of the study. The remaining authors declare that the research was conducted in the absence of any commercial or financial relationships that could be construed as a potential conflict of interest.
